# The First Case of L. pseudomesenteroides Pulmonary Infection and Literature Review

**DOI:** 10.1155/2020/8818491

**Published:** 2020-11-17

**Authors:** Liwan Dai, Yishi Li, Xiaobing Zhang, Ming Ding, Shuliang Guo

**Affiliations:** Department of Respiratory and Critical Care Medicine, The First Affiliated Hospital of Chongqing Medical University, Chongqing, China

## Abstract

L. pseudomesenteroides is a very rare bacterium that infects human beings, and it has been used as an industrial fermentation bacterium. At present, only a few cases have been reported about this bacterium infecting the human body, but most reports are mainly about sepsis. We will report on a woman with lymphoma who was successfully diagnosed by the use of transbronchial cryobiopsy (TBCB) with L. pseudomesenteroides pulmonary infection.

## 1. Instruction

L. pseudomesenteroides is one of the species of Leuconostoc. An infection caused by Leuconostoc is very rare [1]. It has long been regarded as a necessary bacteria in the production of dairy products, fermentation, brewing, and sugar industry and is not a common pathogen [2]. Since 1984, Lee et al. reported for the first time the infection caused by Leuconostoc, and there have been few reports of human infection [3]. Recently, we have a case of lymphoma patients with diffuse pulmonary infiltration of lesions; lung biopsy used by transbronchial cryobiopsy (TBCB) got satisfactory specimens. In the lung tissue, we cultured L. pseudomesenteroides and confirmed its presence by mass spectrometry. As we know, this is the first reported infection of L. pseudomesenteroides to have been identified in live lung tissues.

## 2. Case Report

**Figure 1:**
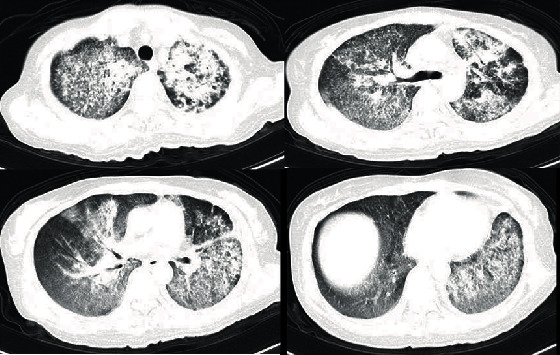
Characteristics of chest CT scan changes in patient.

**Figure 2:**
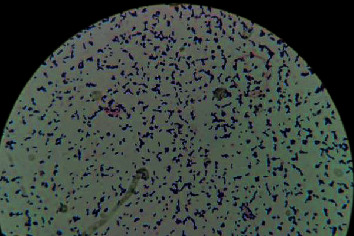
L. pseudomesenteroides and microscopic image. Arrows indicate blue-purple changes in Gram stain microscopically, a full field of it.

A 55-year-old woman was admitted to a hospital due to upper abdominal pain 5 years ago. Physical check-up revealed superficial lymph nodes enlargement; lymph node biopsy was taken and pathologically diagnosed as “diffuse large B cell lymphoma.” According to the course of treatment, “NCID, NCOD, NCID, ECHOP, ECHOP, and CHOP” chemotherapy regimens were given 6 times, the treatment effect is good, and the condition was controlled; then, she regularly attended the follow-up thereafter. 1+ years ago, there was progressive enlargement of the left cervical lymph node; she was considered for recurrence of lymphoma; she was treated with RCHOP and CHOP regimens twice again. After chemotherapy, her white blood cells decreased and remained between 2 and 3^∗^10^9^/L for a long time. 4 months ago, the patient had recurrent low fever without obvious inducement and hyperthermia after the activity occurred. 1 month ago, she took a chest CT scan; the CT demonstrated diffuse distribution of microscopic nodules and specular ground glass density shadows and consolidation shadows in both lungs, with blurred edges, and the subpleural area was relatively less affected; multiple calcified lymph node shadows were observed in the mediastinum and hilum of both lungs; and there was a slight effusion in the bilateral thorax (Figure 1). We admitted the patient to inpatient hospital for fourth diagnosis and treatment. After admission, the temperature fluctuation was found to be 38°C-39.5°C, routine blood test indicated that the white blood cells fluctuated between 1.8 and 2.5^∗^10^9^/L, and two blood cultures were negative. Bone marrow puncture suggested that bone marrow hyperplasia was not active and tissue cells 2%. After admission, we improved sputum culture for the patients many times, all of which were negative, but the patient was considered to have the basis of low immunity, combined with hematogenous system tumor; we considered that the infection caused lung lesions. Cefoxitin, imipenem/cilastin sodium, vancomycin, and voriconazole were successively given. But the temperature has not returned to normal and the symptoms of dyspnea are still serious to determine the etiology of diffuse lesions. In order to determine whether the patient's lung was mainly manifested by infection or lymphoma metastasis, we decided to conduct bronchoscopic frozen lung biopsy for the patient. On January 8, 2016, transbronchial cryobiopsy was performed under a general anaesthesia. Alveolar lavage fluid was collected for culture before biopsy. The results of alveolar lavage fluid indicated that the microbial culture was negative, and no lymphoma cells were found. The pathological results showed that granulomatous inflammation, necrotic material, and a few glassy tissues were observed. Immunohistochemical findings include CK (-), EMA (-), CD68 (+), Ki67 5% (+), PAS (-), CD45 (+), CD20 (-), and CD3(+). Lymphoma pulmonary infiltration was excluded. After 5 days,lung tissue culture suggested L. pseudomesenteroides and (Figure 2), Streptococcus sanguis, and Enterococcus faecalis. Subsequently, we asked the clinical laboratory to determine the type of bacteria again; they used mass spectrometry to reconfirm the type of bacteria. Pseudomesenteroides is naturally resistant to vancomycin. Penicillin is the first choice for treatment. Because the patient continued to use vancomycin at 500 mg q12h^∗^10 days, imipenem/cilastin sodium 1 g q8h^∗^12 days, and voriconazole 200 mg q12h^∗^7 days until the culture results were available, we decided to continue treatment with penicillin 1.92 g q8h and voriconazole. Unfortunately, the patient died of severe respiratory failure followed by multiple organ failure even after the medication was changed.

## 3. Discussion

Leuconostoc is a Gram-positive facultative anaerobic coccus or coccobacillus, catalase, and oxidase negative, which grows in pairs and chains, forming colonies morphologically mistaken for Enterococcus or Streptococcus viridans by routine biochemical testing in the clinical microbiology laboratories [4]. It is composed of Leuconostoc mesenteroides and 7 other species, L. gelidum, L. carnosum, L. fallax, L. citreum, L. argentinum, L. pseudomesenteroides, and L. lactis [5, 6]. It is commonly found in the dairy fermentation, brewing, and sugar industry [7, 8] and is occasionally isolated from gastric, intestinal, and vaginal secretions [9]. Leuconostoc is intrinsically resistant to vancomycin because of its pentapeptide cell wall precursors ending in a depsipeptide (alanine-lactate) rather than in the alanine-alanine dipeptide, which is the binding site for vancomycin in susceptible Gram-positive cocci [4]. In the past, Leuconostoc was not thought to be pathogenic to humans, but occasional cases of infections caused by this organism [10] such as ventriculitis [11], osteomyelitis [12], and bloodstream infection especially bacteremia is more common in case reports [4, 9–14]. The specific mechanism of the disease is not clear; risk factors for infection by Leuconostoc include central venous catheters, parenteral nutrition, surgery, liver failure, chronic renal insufficiency treated with hemodialysis, extensive burns, compromised immunity, and previous antibiotic therapy, particularly with vancomycin [9, 15].The skin and digestive tract are believed to play important roles as routes of entry into the body [2]. Vancomycin therapy and long-term intravenous nutrition, through a central venous catheter, might have played some role in the development of Leuconostoc bacteremia in this patient [13]. There are no standards for selecting antimicrobial agents to treat Leuconostoc. The treatment of choice seems to be penicillin or ampicillin, but clindamycin, linezolid, macrolides, aminoglycosides, cephalosporins, and tetracyclines have also been used [9, 11, 12]. In this case, the patient had a lymphoma basis, a low level of white blood cells for a long period of time, low immunity, and host factors associated with infection.

Secondly, for the collection of patient samples, we adopted the world's advanced lung biopsy method, transbronchial cryobiopsy. Transbronchial cryobiopsy has recently been proposed as an alternative to surgical biopsy in the diagnosis of diffuse lung disease [16]. Performed correctly, it appears to have a better safety profile than surgery [17]. In this way, through the use of bronchoscopy, we can understand the monitoring of the bronchoscope, into the lobe segment corresponding to the chest CT lesion; and then, the frozen probe was used to take a biopsy of the lung tissue at the designated site; this ensures the accuracy of the sampling. The tissue sample is pollution-free, and the cultured bacteria should be the bacteria growing in the patient's lung. The result was the coexistence of three pathogenic bacteria: L. pseudomesenteroides, Streptococcus sanguis, and Enterococcus faecalis; because of forming colonies morphologically mistaken for Enterococcus or Streptococcus viridans, we used mass spectrometry [5, 6, 18] to identify the strains and obtained the results. Our patient was treated with vancomycin and imipenem/cilastin sodium after admission; the antibacterial spectrum of these drugs has covered Enterococcus faecalis and Streptococcus haematococcus. On the contrary, the natural resistance to vancomycin may further aggravate the imbalance of bacteria which may have been caused by the excessive growth of L. pseudomesenteroides.

Thirdly, the patient had a basis of lymphoma. During the diagnosis, the patient should be paid special attention to whether her lung infection was caused by pulmonary infiltration of lymphoma. The pathologic result was granulomatous changes which was not consistent with the characteristics of lymphoma. At the same time, the immunohistochemical CD20 was negative, but the slight positive change from CD3 was not enough to diagnose lymphoma, so the possibility of lymphoma pulmonary infiltration was ruled out. It proved that both lung changes are caused by infection.

Finally, unlike other reported cases, our patients did not use deep vein catheterization or parenteral nutrition except for routine infusion with indwelling needles. No pathogenic microorganisms were detected in peripheral blood culture. It is not consistent with the possibility of bloodborne infection as previously reported in medical records. So, whether this kind of bacteria has other ways to infect the human body remains to be further explored.

## 4. Conclusion

Leuconostoc is not considered to be a usual part of the normal human microbiological flora [3]; low immunity and critically ill patients are at high risk of this pathogen [2, 3, 9, 10]. In particular, the identification of drug-resistant bacteria during the use of vancomycin should be emphasized. The detection of biopsy in living lung tissues provides a new idea for us to understand the pathogenic process of this bacterium. Transbronchial cryobiopsy (TBCB) has also been shown to be an effective way to assist clinicians in diagnosing diffuse lesions, even in critically ill patients; this way can still be operated on a ventilator support. Perfecting pathological biopsy as soon as possible can help to improve the treatment rate of patients and reduce the error rate of empirical treatment.
